# Experiences with developing and implementing a *courageous conversations* pilot classroom through synchronous meetings via zoom

**DOI:** 10.1017/cts.2022.488

**Published:** 2022-10-28

**Authors:** Cilia E. Zayas, Tremaine B. Williams, Mathias Brochhausen

**Affiliations:** 1 Department of Biomedical Informatics, University of Arkansas for Medical Sciences, Little Rock, AR, USA; 2 Department of Medical Humanities and Bioethics, University of Arkansas for Medical Sciences, Little Rock, AR, USA

**Keywords:** Education, diversity, equity, inclusion, biomedical informatics

## Abstract

**Background:**

The murder of George Floyd created national outcry that echoed down to national institutions, including universities and academic systems to take a hard look at systematic and systemic racism in higher education. This motivated the creation of a fear and tension-minimizing, curricular offering, “*Courageous Conversations,”* collaboratively engaging students, staff, and faculty in matters of diversity, equity, and inclusion (DEI) in the Department of Health Outcomes and Biomedical Informatics at the University of Florida.

**Methods:**

A qualitative design was employed assessing narrative feedback from participants during the Fall semester of 2020. Additionally, the *ten-factor* model implementation framework was applied and assessed. Data collection included two focus groups and document analysis with member-checking. Thematic analysis (i.e., organizing, coding, synthesizing) was used to analyze a priori themes based on the four agreements of the *courageous conversations* framework, stay engaged, expect to experience discomfort, speak your truth, and expect and accept non-closure.

**Results:**

A total of 41 participants of which 20 (48.78%) were department staff members, 11 (26.83%) were department faculty members, and 10 (24.30%) were graduate students. The thematic analysis revealed 1) that many participants credited their learning experiences to what their peers had said about their own personal lived experiences during group sessions, and 2) several participants said they would either retake the course or recommend it to a colleague.

**Conclusion:**

With structured implementation, *courageous conversations can be an effective approach to* create more diverse, equitable, and inclusive spaces in training programs with similar DEI ecosystems.

## Introduction

On May 25, 2020, the wrongful death of George Floyd, a 46-year-old Black man murdered by a White police officer in Minneapolis, Minnesota sparked a national outcry [1]. Demonstrations erupted throughout the USA after graphic video clips of his death went viral on social and broadcast media [2]. Protests, however, were not limited to the USA. Hundreds of thousands of people gathered in cities throughout the world to strike against Floyd’s death, demonstrating that the Black Lives Matter campaign (a grassroots movement in the USA created in 2013 to combat racial inequity and police brutality) [3] was resonating with broader demands to contest and counter racism on a universal scale. Floyd’s death became a symbol for global racial inequality with protests ushering a multinational reckoning about the ways that institutions, policies, and cultural norms oftentimes benefit Whites and disadvantage people of color.

This national outcry was heard, and a call to action echoed down to national institutions, including universities and academic systems to take a hard look at systematic and systemic racism in higher education [4]. On June 10, 2020, thousands of academics and scientific research institutions worldwide entered a call to “Strike for Black Lives,” suspending their daily work practices to learn about structural inequality in the scientific world and to design interventions to address inequalities [5]. In response, University of Florida President, Dr Kent Fuchs, strongly encouraged the academic community to, stop for a day to reflect upon the social unrest that had gripped the nation, commit to educate ourselves about racism, in particular anti-Black racism, and pursue change [6].

Acting in response to this call to action, the authors reflected on the role of curriculum in fostering diversity, equity, and inclusion (DEI), particularly at the department level. Based on domain-related research in DEI, an initial draft of the curriculum for a DEI-based, c*ourageous conversations’* classroom was developed in collaboration with education leadership in the Department of Health Outcomes and Biomedical Informatics (HOBI) at the University of Florida. The course’s syllabus was grounded in Singleton & Linton’s (2006) protocol on *courageous conversations* [7]. Implementation was guided by Pinto & Slevin’s (1987) *ten-factor model of the project implementation process* to manage and assess the operationalizing of the protocol [8]. Thus, the primary purpose of this project was to report a student–faculty collaborative approach to developing a pilot *courageous conversations’* classroom for HOBI faculty, students, and staff. Recommendations for student and faculty course facilitators are provided. The purpose of the project was guided by the following objectives:

- Replace silence with open dialog between students, staff, and faculty on topics that are often difficult, uncomfortable, and in many cases, painful.
- Utilize existing DEI literature and personal narratives reflecting the lived experiences of those suffering from the lack of DEI as the basis for the discussions.
- Search for implementable ideas that can be introduced at the university and or department to make the research community more diverse, equitable, and inclusive.
- Explore and develop a *courageous conversations* classroom model as a course for students that could be accessible to faculty and staff in future semesters and replicated to institutions with similar DEI ecosystems.

## Methods

A qualitative design was employed assessing narrative feedback from participants during the Fall semester of 2020. Additionally, the *ten-factor* model implementation framework was applied and assessed. We utilized a multifaceted approach to collecting data: a web-based, google document and two focus groups. Web-based, google documents were uploaded to the e-learning site in advance of the debriefing sessions for participants to provide comments. Additionally, two focus group sessions were used to facilitate open discussions debriefing items during the final two sessions. The web-based, google document remained visible to all participants through zoom’s share document feature. We performed real-time member checking during the focus groups to ensure collected data was interpreted as the participant intended. Participants who were uncomfortable providing written or verbal feedback within the group setting were encouraged to the facilitators to share their experiences. Thematic analysis (i.e., organizing, coding, and synthesizing) was used to analyze a priori themes based on the four agreements of the *courageous conversations* framework, stay engaged, expect to experience discomfort, speak your truth, and expect and accept non-closure. The analysis was completed within anonymized documents that were posted to the e-learning platform in advance of the final two group debriefing sessions. Documents provided an unstructured opportunity for participants to share feedback related to their experience within course. This promoted the participant’s ability to freely express their thoughts regarding; the feelings the course may have evoked, the content of the discussion material, and areas of improvement.

### The Four Agreements of Courageous Conversations

Openly examining and addressing topics of inequality, discrimination, and lack of inclusion is often difficult, not just intellectually but emotionally. To bring faculty, staff, and students together to candidly examine and discuss such topics, we adopted a framework by which to engage with one another in a safe and nonthreatening environment. We chose the “Four Agreements of Courageous Conversations” [7] to facilitate discussions and keep them flowing in a respectful and mindful manner. *Courageous conversations* are a technique that aims to minimize the tensions and fears that often surround discussions about race, gender, gender identity, and the injustice of inequality by creating an environment that allows those who have lived experiences on these particular topics the opportunity to share them and for those who do not have such first-hand knowledge to develop and grow from such shared experiences and subsequent conversations. *Courageous conversations* consist of a set of agreements that challenge tightly held cultural norms to steer clear of race talk or avoid examining and rectifying organizational barriers that may hamper the success of students, faculty, and staff based on their race, gender identity, and/or ethnic background. The whole group engaging in *courageous conversations* commits to the “Four Agreements of Courageous Conversations”: to stay engaged, expect to experience discomfort, speak our truth, and expect and accept non-closure.

#### Stay engaged

Staying engaged was defined as “remaining morally, emotionally, intellectually, and socially involved in the dialogue” (Singleton & Linton, 2006, p.59). As part of meeting this agreement group participants were asked to hold themselves individually accountable as to their level of engagement even when the conversations feel uncomfortable.

#### Expect to experience discomfort

This agreement recognizes that discomfort is unavoidable, especially in discourses, touching on race/ethnicity, gender, sexual orientation, and the resulting discriminations and inequalities, but participants are committed to bringing such issues to the open. It is not the practice of honest and truthful engagement with these conversations that creates division. The division already exists within workspaces and within our society. Ignoring them is the problem. Through conversation, even when they become uncomfortable is when healing and change occurs. This is a key agreement because the intervention or the catalyst for change that the pilot *courageous conversations* classroom aimed to foster was to embrace such dialog and not steer clear of it.

#### Speak your truth

This agreement asks participants to speak openly about their lived experiences or honestly convey their message. Singleton & Linton (2006) proposes that participants use “I” statements to do so. While, speaking one’s truth is valued and valid any perceived truths that were expressed in a manner that was harmful, demeaning, or disrespectful were not tolerated and were addressed by group facilitators.

#### Expect and accept non-closure

This agreement asks participants to expect and accept it will take time and will require an ongoing dialog, especially, in relation to racial understanding and inequities in its many forms. The pilot program aimed to foster *courageous conversations* among students, faculty, and staff surrounding pervasive inequalities and lack of diversity in academia as a first step to reach improved understanding in these matters and thereby create effective restorative solutions. Expectations that solutions would be identified and implemented swiftly were revised among group members.

### Classroom Norms and the “Circle of Trust”

To enable a group of faculty, staff, and students of one department to engage in the “Four Agreements of a Courageous Conversation” classroom fully and successfully, norms had to be formulated and established. These norms were referred to as community guidelines within the pilot course. It was also emphasized that the aim of these discussions was not to “score a point” but rather to provide a space for sharing, conversing, and thinking about our truth. As part of these guidelines, facilitators would make every effort to create and foster a classroom environment that reflected respect and empathy and was conducive to sharing and listening to narratives and discussing these often-uncomfortable topics. The approach used to create such an environment of safety and trust was based on a system of contemplative talking circles, also known as “circles of trust,” developed by Parker J. Palmer, Founder, of the Center for Courage and Renewal [9].

Participants were also invited to contact facilitators and suggest how their role as facilitators could be made more active or proactive in creating a safe space. We also openly addressed the topic of fear of retaliation with group participants. Classroom sessions would not be recorded for this reason. Taking away the video freed people to stop concentrating on whether such footage might resurface or be used against them, and instead focus more on the content of the sessions and dialog. The emphasis is building trust among colleagues when discussing difficult topics. It is also recognized that silence should not be perceived as lack of engagement while discussing topics that are difficult and invoke discomfort. We, therefore, provide a space for individuals to listen and learn from others lived experiences. When exposed to topical discussions that can elicit an emotional response silence could also be interpreted as a function of self-reflection. As such, the facilitators chose to ask students for self-assessment of their level of engagement rather than assessing each participant’s engagement by visible behaviors. These are recommendations of Singleton & Linton in dealing with and interpreting engagement in this context.

### Discussion Topics

We divided the discussion topics throughout the semester into six categories: Antiracism; Feminism; Women in academia; Structural racism and health inequities; Microaggressions in academic medical environments; and Lesbian, Gay, Bisexual, Transgender, and Queer plus (LGBTQ+) persons in Science, Technology, Engineering, and Math (STEM). In addition to these thematic categories, we planned for a multi-session debriefing section. Readings and videos included learning about the recent history of racism in the USA, in Ibram X. Kendi’s *Stamped from the beginning: the definitive history of racist ideas* (2016) [10] and *How to be an antiracist* (2019) [11]. As well as how the interrelated nature of race, class, gender, and other individual characteristics create overlapping layers of discrimination were discussed via Kimberlè Crenshaw’s work on intersectionality [12], and articles about social and environmental justice leaders and grass roots organizations who made changes in their communities, such as Dr Robert Bullard [13], and Alexa Ross, co-founder of Philly Thrive [14]. A copy of the complete *courageous conversations* course syllabus is provided in the Appendix.

We employed a broad recruitment strategy, inviting all student, faculty, and staff members of the department to participate in the pilot course. Individuals would participate on a voluntary basis. Multiple invitations to register for the pilot course prior to the registration deadline was delivered via email. A flyer was created and included in the email to highlight the invitation and differentiate from the multiple emails department members receive throughout the day. A copy of the marketing flyer that was included in the email is provided in the Appendix. No cost was assigned to the pilot program to eliminate financial barriers that could limit participation. It was also important to ensure maximum participation of students and staff who work full-time or part-time positions. We solicited the help of senior leadership to allow working students and staff members to participate in the pilot course, if they so desired, during work hours. Individuals wanting to participate would express their interest to their respective managers, and the managers were advised by senior leadership to make all concessions possible to participants to join the pilot course without retaliation. This change in policy to encourage participation in the pilot course was marked by emails sent from the HOBI leadership team to the entire department.

Aggregated data, including enrollment figures, reflecting the percentage of faculty, students, and staff participation in relation to the entire department are provided. Other data collected were anonymized debriefing documents that allowed group members to provide feedback for the thematic analysis. The last data type we present is participant engagement with course resource materials as indicated by the UF e-learning platform. E-learning includes a mechanism called *New Analytics* by which course facilitators could gauge page views, which represents the count of hits for a particular page in the module. Modules were created with pages for uploaded videos, readings, and documents that included reflection questions for the group sessions described in the results section.

### Implementation Process

We adopted the *ten-factor model of the project implementation process* to guide the implementation of the *courageous conversations* pilot program [8]. The model provides a list of 10 empirically derived critical success factors that should be considered to improve the chances of project implementation success [15]. Each critical factor was incorporated in the design of the implementation strategy. Table 1 summarizes how we operationalized each factor throughout the implementation process.

**Table 1.** This is how the implementation strategy matches up to the framework’s critical factors for successful project implementation

Critical Factor	Definition	Comments
Project mission	Goals and general direction of project are initially clear	Graduate student, Cilia Zayas, formulated the mission, prior to presenting the idea and motivation for implementing a *courageous conversations* classroom to top management for support. The graduate student reviewed online resources, journal articles, textbooks, and podcasts on how to create a mission and develop an actionable plan in support of this effort. Meaningful resources were provided by *Particles for Justice* [16], *#shutdownSTEM, and #shutdownacademia* [17]. As well as The Equity and Inclusion Journal Clubs at Harvard University, and University of Pennsylvania, respectively [18,19]. These resources were then summarized, and the mission, goals, and general direction of the pilot class unique to the Health Outcomes and Biomedical Informatics department were developed.
Top management support	Top management’s willingness to provide the requisite support and authority/power to ensure project success.	Graduate student presented the idea and motivation during a Diversity, Equity, and Inclusion Committee meeting. Gained Department and Education Leadership support. Support for the program was provided verbally and in written form by leadership team. Multiple emails were provided to department employees and students that participation in the pilot program was permissible during work hours. Accommodations would also be made available to the extent possible by team managers for individuals wanting to attend.
Project schedule or plan	A thorough description of each of the project’s individual action steps.	Developed draft course syllabus as part of student/faculty collaboration.
Client consultation	All parties affected should be informed, consulted and actively listened to.	Education leadership presented the idea and draft course syllabus to department faculty, staff, and students for feedback. An invitation to join the pilot program’s design and implementation team as a co-facilitator was extended to administrative staff members. Feedback was favorable. Some faculty members provided editorial comments to draft syllabus. Certain department staff verbalized their desire to join the co-facilitator team but were concerned that existing work schedules would not permit them to take on such responsibility.
Personnel	Recruitment, selection, and training of the project team’s necessary staff.	Personnel in the department’s communication team were consulted to help design a marketing flyer for the pilot program, and the education department administrative specialist offered her time to assist with logistics, and registration of participants to the online e-learning platform.
Technical tasks	The required technologies and expertise to complete the specific technical action steps are available.	Responsibilities were divided between design and implementation participants. Create marketing campaign (flyer of pilot emailed) with deadline to register. Register students. Create and distribute email with google poll to all faculty, students, and staff to determine the best day and time for meetings. Date with highest number of respondents was selected. Discussion of feasibility of using the student-centered e-learning platform used in HOBI’s academic programs. Create e-learning platform modules for course material. Email meeting invite with recurring zoom link for registered participants.
Client acceptance	The act of “selling” a completed project to its final consumer.	Was determined based on registered participants who attended weekly classroom meetings. The pilot courageous conversations classroom included a total of 41 participants of which 20 (48.78%) were department staff members, 11 (26.83%) were department faculty members, and 10 (24.30%) were graduate students.
Monitoring and feedback	At each point of the implementation process, timely and detailed control information is provided.	At each stage of the implementation process key personnel provided status reports and received feedback on project timeline, including making sure everything was in order for class start date, Friday, September 25 at 11:00 a.m. EST.
Communication	All main players in the project’s implementation will have access to a suitable network and the necessary data.	Communication was maintained between all facilitators via scheduled zoom meetings, email correspondence, and phone calls.
Troubleshooting	Ability/capacity to deal with unforeseen crises and deviations from the original plan.	Developers and co-facilitators managed unexpected deviations from initial plan. Example of this was the need to address miscommunication regarding the closed cohort nature of this group experience. Problem was resolved by directly addressing parties affected and sending out a final call for individuals who wanted to participate but failed to register by the initial registration deadline.

### Post-Implementation Strategy to Ensure Project Success

Facilitators met before the session started on multiple occasions to plan administration, implementation, and course content. They also met for 1 hour after weekly classroom meetings to discuss and debrief regarding the current session, plan the following lesson, and discuss opportunities that could be created by amending the current plan. Frequently, facilitators sent out emails to ask the class if they would like to revisit a topic that was not fully covered in the previous session.

## Results

The pilot *courageous conversations* classroom was implemented at the Department of Health Outcomes and Biomedical Informatics at the University of Florida. It was a voluntary educational offering that was advertised to all members of the department and supported by Department and Education Leadership. The *courageous conversation* classroom met weekly for 1 hour. Course information, including readings, links to videos, and syllabus were made available through the campus e-learning online platform. Materials were posted 5 days in advance to allow participants time to review and consider questions for discussion. The meeting was divided so that 45 minutes were dedicated to dialog and exchange and 15 minutes to discuss suggestions geared towards departmental policy changes participants would like to see implemented because of the conversations and materials exchanged.

The facilitators were as follows: one PhD student, Cilia Zayas, and one tenured faculty member, Dr Mathias Brochhausen, who also served as the Co-Director of Education. While the primary goal of the facilitators was to meet the pilot program’s four objectives, per the curriculum (see Background) in collaboration with all participants, we also aimed to assess how well the group followed the “Four Agreements of Courageous Conversations” (i.e., 1.) *stay engaged*, 2.) *expect to experience discomfort*, 3.) *speak your truth*, and 4.) *expect and accept non-closure)* as part of this semester long, team building, group exercise. In addition, we relied on the Circle of Trust® approach to meet each of the four objectives.

The following sections summarize participation and provide a) a qualitative assessment of the pilot program from the perspective of the two facilitators and b) the perspective that the participants shared with the group.

### Participation

**Fig. 1.**
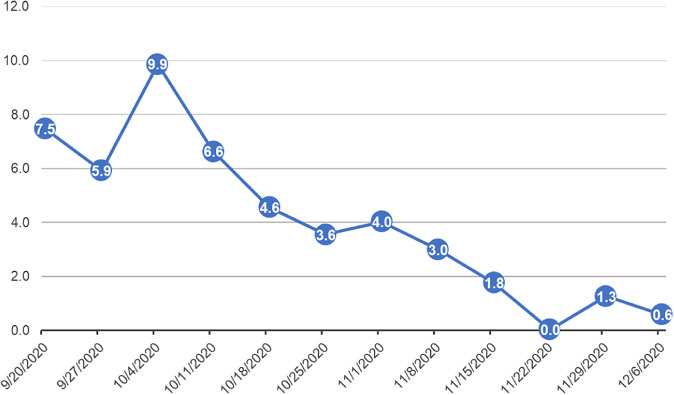
Average page views.

Fig. 1 indicates weekly user interactions with course resources. The *x*-axis is the start date of the class week. The *y*-axis is the number of page views, which represents count of hits for a specific page. The points in the line graph represent the average page views by the total number of participants enrolled. For instance, during the week of 09/20/2020, there were a total of 284 page views among 38 participants with an average page views of 7.5. These figures were provided as course analytic reports from the e-learning platform. It should be noted that this figure excludes page views from both course facilitators and the e-learning administrator. Although, course facilitators and e-learning administrator were participants in the pilot course, we exclude their respective pages views because these individuals entered pages to create the modules and edit the platform. The number of page views would have been inflated had we included these counts. The average page views represented in Fig. 1 capture participant interactions with the resource materials in preparation for or as follow-up to meeting discussions.

The *x*-axis is the start date of the class week. The *y*-axis is the number of page views, which represents count of hits for a specific page. The points in the line graph represent the average page views by the total number of participants enrolled.

Page views as illustrated in Table 2 is defined as the count of hits for a particular page in the module. Modules were created with pages for uploaded videos, readings, and documents including reflection questions for the week’s session. Participants could access content multiple times.

**Table 2.** Page views by module

Modules	Page views (count)
Week 1: Introduction	99
Week 2: Stamped from the beginning	174
Week 3: How to be an anti-racist	139
Week 4: We should all be feminists	146
Week 5: Turning chutes into ladders for women faculty	39
Week 6: Structural racism and health inequities in the USA	82
Week 7: Microaggressions	67
Week 8: Queer in STEM	54
Week 9: Group debriefing	16
Week 10: Group debriefing	18

Last, Table 3 below provides the percentage of faculty, student, and staff who participated in the pilot course in relation to the entire department.

**Table 3.** Faculty, student, and staff participation in relation to total department

Position	Department	Pilot course	% Participation
Faculty	31	11	35.48%
Student	38	10	26.32%
Staff	111	20	18.01%
Total	180	41	22.78%

### Facilitator Themes

#### Stay engaged

Facilitators asked participants to self-evaluate the degree to which they remained engaged during the weekly conversations (i.e., in lieu of facilitators measurements of engagement). Participants self-identified and monitored moments when they drifted from the topic of conversation and introspectively asked “why” to recommit their attention for the remaining class time. The most significant indicator of engagement was met that each week the group discussions would last the full 1-hour session. Consistently, the facilitators allowed the sessions to run past their scheduled times. The facilitators heard from a variety of participants each time, and for many of the weekly sessions’ participants felt safe enough to share intimate and personal realized experiences that were often difficult to hear. Also, for those, who found themselves doing more listening than sharing, they often openly expressed their empathy and resolve to reduce systemic racism and disparities that exist in our shared work environment. It is understood that in discussing issues that are difficult and invoke discomfort silence should not be interpreted as lack of engagement. Therefore, the facilitators chose to ask students for self-assessment rather than assessing each participants engagement by observable behavior. It was evident that many of the participants listened intently, asked questions, responded to questions, and reacted to both the materials and their colleagues expressed lived experiences.

#### Expect to experience discomfort

The facilitators presented the objectives to all participants on the first day of class and underscored the fact that as a group we were going to intentionally engage with material and discuss topics that would bring about uncomfortableness. Singleton & Linton (2006) identified this element as necessary to pursue *courageous conversations* about forms of injustice and inequality. There were many times the facilitators personally felt uncomfortable with the material or conversations. The pre-class sessions provided a useful format to discuss moments of discomfort and how to handle them between the facilitator group. The discomfort, however, served as a bridge for personal growth and self-education on the topics of discrimination, equality, and integration.

#### Speak your truth

Speaking one’s truth can be scary and therefore takes tremendous courage. Hence, speaking one’s truth can be a challenge in a group setting. Part of that challenge is that being vulnerable with those who have similar experiences is different from being vulnerable in a space with others who may have completely different lived experiences. This is particularly true when discussing social matters, such as racism and inequality in its many forms (race, ethnicity, gender, gender expression, and ability). The facilitators observed that the fellow participants consistently used “I” statements when expressing experiences that often took valor to share in a group forum. Achieving this was not immediate, however. As facilitators, it was the responsibility to tactfully point out when participants veered away from using “I” statements and reverted to a platform of debating topics or critiquing ideas.

Because the facilitator team included representatives of the student body and faculty, the ability to emphasize the importance of the framework (speak your truth and use “I” statement when doing so) was easier to implement. This also helped balance power differentials. For instance, when members of faculty failed to use “I” statements or were more apt to debate, having a tenured faculty member as part of the facilitator team made it easier to remind participants or certain individuals of this behavior. While the PhD student who was also part of the facilitator team was supported and emboldened to also do so, we understood that we did not want to place any undue pressure on the graduate student facilitator when it came to addressing such matters with participants. The goal was not to police the conversation but rather to reiterate that the discourse was not centered around “scoring a point,” but rather listening and sharing for growth at the individual and group level.

#### Expect and accept non-closure

The facilitator team helped frame such expectations by sharing in a truthful and honest fashion with all participants on the first session together that it was not realistic to implement immediate change on many of these topics. Having these courageous conversations with members of the faculty, administrative staff, and study body was a first step in the right direction, however. Facilitators also felt that leaving the last two class sessions open to create a group document that addressed open-ended concerns for future discussions and proposed solutions helped with this agreement.

### Participant Themes

To gauge participant perspective on this collective group exercise to engage in *courageous conversations* related to DEI, we created two group debriefing sessions at the end of the semester that would allow space and time for participants to share their thoughts about their experience. To facilitate these sessions, we created google documents and posted them to the e-learning platform in advance of each meeting. These documents would allow group members to share their respective thoughts in a completely anonymous way. We did not provide a structured format as to how and what participants should write. Instead, we encouraged members to write comments that could touch upon several related topics including, but not limited to, what they learned and felt during the class time together, the subject matter content, and ideas for improvement. As facilitators, we then began each of these sessions by reading through the comments in the document. We then asked participants if anyone would like to expand on what was written, and for those who did not have a chance to include comments that they would be welcomed to openly share during the group session. The facilitator team took notes, while group members either expanded on their written points or volunteered new material for inclusion in the google documents. These notes were reviewed by the group as they were taken. In the next section, we summarized shared participant perspectives.

#### Stay engaged

In the debriefing sessions, several participants expressed that they enjoyed the course offering and the knowledge gained not only from the materials but also from what was shared by colleagues. Others described the course as being extremely informative and the discussions profound. When asked if anyone would either retake the course or recommend it to a colleague, multiple participants responded yes to each. These comments indicate a certain level of engagement among participants as they appeared intent on learning from each other and committed to think deeply about the subject matter.

#### Expect to experience discomfort

The degree of such discomfort created through *courageous conversations* is personal and topical. In the debriefing sessions, no participant outwardly expressed their discomfort with any given topic or session, but there were instances when group members reacted to either the readings, videos, or others lived experiences in a manner suggesting discomfort for what was being covered in a particular session. This was expected. We did not require participants to vocalize their moments of discomfort or why they felt that way.

#### Speak your truth

Examination of the debriefing documents indicated several participants attributed their learning experience to what their colleagues expressed as their personal lived experiences in the group meetings. This suggests that participants acknowledged when others spoke their truth and the use of “I” statements personalized the message for those listening.

#### Expect and accept a lack of closure

The *courageous conversations* protocol asks participants to expect to be in a state of uncertainty and to not expect immediate fixes to many long-standing injustices often experienced by people of color, women, and LGBTQ + at all levels of academia and research. Many of the group participants embraced the idea that the first steps towards creating meaningful solutions and influencing policy at the department level began with first engaging in these types of conversations. Debriefing documents indicate participants desire to see this course offered again and would themselves retake the course in the future. Many also felt that many of the recommended solutions discussed throughout the semester should be documented and followed up with to push meaningful change. For instance, members recounted in the debriefing document the session on microaggressions and suggested that the department consider creating a mechanism by which microaggressions could be reported anonymously.

## Discussion

Broadly, the pilot of the courageous conversation protocol emphasized the value of integrating the lived experiences of students and colleagues into learning environments. An institution could possess the most scientifically robust curricula and faculty; nevertheless, fail students and faculty of underrepresented groups through a lack of pedagogical designs that account for the racial identities and lived experiences needed to effectively engage [20,21]. The efficacy and effectiveness of teaching strategies that address the effects of cultural discontinuity (i.e., multiculturalism, systemic racism, etc.) as well as interacting with students of color within their classrooms have been assessed for over a quarter of a century [22–24]. Of the robust pedagogical approaches, the pilot demonstrated the effectiveness of the *courageous conversations* protocol as a mechanism for supporting the instructional needs of students of color. With wider-scale adoption, this protocol could reform the structural and procedural components of the current Eurocentric educational systems to mirror the cultural (i.e., racial, gender, and social) diversity that is increasingly reflected in the academic environments of US higher education instructions.

### Limitations

Personal characteristics of the participants were not collected (i.e., age, race, gender they identify with, or political affiliation) to protect the integrity and tonality of the discussions. The course facilitators felt that asking people for personal demographic information they may want to keep private could negatively affect participation [25,26]. Additionally, we acknowledge the limitation of self-selection as a by-product of our recruitment strategy. Participants who voluntarily enrolled in the course based on identifying with the ideologies of Ibram X. Kendi on race in America are likely to be very different from the participants who enrolled that identify with the ideologies of the writings of Clarence Thomas or commentaries of Pennsylvania senate contender Kathy Barnette. As such, we were not able to assess differences in motivation for engaging in the course within the fuller spectrum of political and sociocultural perspectives that exist within the USA. However, this limitation should motivate future inquiry. This course was a pilot. Therefore, as the course is continually offered within our institution and at other institutions with different DEI ecologies throughout the nation, individuals with different backgrounds, beliefs, and perspectives will test the course as a potential, national solution for facilitating DEI within training programs.

## Conclusion

The open conversations help with building an environment that is less frightening and more likely to identify department level allies. Neither a journal club format nor an extracurricular event/opportunity on these necessary but sensitive topics would provide such open and honest dialog, or the commitment to remain engaged in all conversations, despite discomfort. A journal club evokes the idea that academic style discourse is preferred, meaning that articles are debated, and theory emphasized, thereby, diminishing the importance of listening and learning from the personal narratives of those who have historically been underrepresented and who have suffered from the lack of diversity, equality, and or inclusion. Extracurricular signals to participants that one can either choose to participate or not, or one can choose to only participate in the sessions that make the individual the least uncomfortable.

*Courageous conversations* are intended to be a team building experience, and all academic departments could benefit from addressing issues of discrimination, equality, and integration as part of their team building efforts. The chosen format of creating a *courageous conversations* classroom setting encourages participants to strip away titles, position, privilege, and power, and not only listen to one another but also empathize, learn, and incite change. This is a critical part as to how departments grow and function and bring about cultural, political, and educational curriculum changes. If the department is too large, then this format could be adapted at the division level.

More rigorous, qualitative, or quantitative evaluation of the *courageous conversation* classroom would need to be conducted during future implementations of the curriculum.

## Supplementary material

For supplementary material accompanying this paper visit https://doi.org/10.1017/cts.2022.488. click here to view supplementary material
