# Supplementary material for: Experiences with developing and implementing a courageous conversations pilot classroom through synchronous meetings via zoom
Source: J Clin Transl Sci. 2022 Oct 28;7(1):e32. doi: 10.1017/cts.2022.488 (PMC9947604; doi:10.1017/cts.2022.488)
Supplement: Supplementary file 1 [file S2059866122004885sup001.pdf]

Department of Health Outcomes and Biomedical Informatics (HOBI)

College of Medicine

University of Florida

Equity and Inclusion in HOBI Journal Club

Pilot Program

Fall 2020

Meetings: Fridays, beginning September 25, 2020, from 11:00 a.m. to 12:00 p.m.

Location: Zoom videoconferencing

Facilitators: Cilia Zayas, MSF, MHA

Ph.D. Candidate in Medical Sciences, Health Outcomes and Implementation Science

Mathias Brochhausen, Ph.D.

Associate Professor and Associate Director of Education

Contact: cilia@ufl.edu

mbrochhausen@ufl.edu

Description: We will meet regularly in the Fall 2020 semester to discuss issues related to equity and inclusion.

Guiding Principles: The practice and culture of scientific research and discovery is not exempt from the pervasive inequalities within society as a whole. The lack of diversity, especially the under-representation of people of color (e.g., Blacks, Latinx, and Indigenous Americans) women and LGBTQ+ at all levels of academia and research – from graduate students, postdoctoral scholars, staff members, and faculty – is a long established and undeniable problem. It is a problem stemming from the systemic racism and sexism deeply rooted in every sector of our society (e.g., employment, healthcare, housing, education). In trying to build a more inclusive scientific community at the University of Florida and the College of Medicine, the goal of the Equity and Inclusion in HOBI Journal Club is to promote honest and truthful discussions and learn from the historical, societal, and organizational existence of inequality.

Community Guidelines: We acknowledge that the topics we are covering in this journal club are often difficult, not just intellectually but emotionally. It is our goal to create a safe space in which everyone feels comfortable sharing their experiences, thoughts and concerns. We know that expressing such personal accounts or even views on diversity and equality can make people feel vulnerable, and requires courage and trust. We ask all participants to respect each other and phrase their contributions with care and empathy. The aim of the discussions in this course are not to “score a point”, but rather to provide a space for sharing, conversing, and thinking about such narratives. We will make every effort to create and foster a classroom environment that

reflects respect and empathy and is conducive to discussing these often-uncomfortable topics in an environment of safety and trust. If you have suggestions of how the facilitators can be more active or proactive in creating a safe place, please reach out to us. Your opinion, feelings, and help are important to us.

Objectives:

1. To replace silence with open dialogue between students, staff, and faculty on topics that are often difficult, uncomfortable, and in many cases painful. We all have different narratives and lived experiences. Let us embrace the differences and respectfully engage in discussion with empathy and care in order to learn from each other.
2. To use the research literature and the personal narratives reflecting the lived experiences of those suffering from the lack of inclusion and diversity as the basis for our discussions.
3. To search for implementable ideas that can be introduced at the University of Florida and the Department of Health Outcomes and Biomedical Informatics to make our research community more diverse, equitable and inclusive.
4. To explore and develop this journal club as a course for HOBI students (that also continues to be accessible to HOBI faculty and staff as a whole) in future semesters.

E&I in HOBI Journal Club meeting dates and suggested discussion topics:

Date	Location & Time	Journal Articles / videos
September 25, 2020	Online<br><br>11:00 – 12:00	**Introduction**<br>• Discuss the motivation behind developing this pilot program<br>• Go over our guiding principles and objectives<br>• Get to know one another before we begin our discussions
October 2, 2020	Online<br><br>11:00 – 12:00	**Video 1: Stamped from the beginning: The definitive history of racist ideas in America**<br>https://www.youtube.com/watch?v=nBFYQu1ZYD0<br><br>In this video, Ibram X. Kendi, author and former assistant professor of African American History at the University of Florida examines the recent history of racism in the United States, including where racial ideas originate and how they propagate.<br><br>**Reading:** Prologue of Kendi's Stamped from the beginning book (pages 1-11)<br>Reading is available in this week's e-learning module and course reserves<br><br>**Additional Videos:** Please watch at least one of the two added videos in addition to video 1 posted in this week's module<br><br>https://www.youtube.com/watch?v=OYGfLM0QQL4<br>https://www.youtube.com/watch?v=BFsNpswLysU<br><br>**Review:** Questions to consider for the discussion
October 9, 2020	Online<br><br>11:00 – 12:00	**Video 1: How to be an anti-racist.**<br>https://www.youtube.com/watch?v=_nje6B80f4M<br><br>In this book talk, Ibram X. Kendi author and former assistant professor of African American History at the University of Florida analyses the many forms of racism and how we can help eradicate them, while also expressing his personal experiences with racism as well.

		**Reading:** Chapters 1 and 2 of Kendi's How to be an antiracist book (pages 1-11)<br><br>**Review:** Questions to consider for the discussion
October 16, 2020	Online<br><br>11:00 – 12:00	**Video 1: We should all be feminists.**<br>https://www.ted.com/talks/chimamanda_ngozi_adichie_we_should_all_be_feminists<br><br>In this Ted Talk Chimamanda Ngozi Adichie not only defines feminism in the 21st century – grounded in awareness and inclusion, but also helps readers of all backgrounds better understand and acknowledge that sexism exists.<br><br>**Video 2:** Kimberle Crenshaw discusses intersectional feminism.<br>https://www.youtube.com/watch?v=ROwquxC_Gxc<br><br>**Reading:** Chapman and Benis (2017). Ceteris non paribus: The intersectionality of gender, race, and region in the gender wage gap.<br><br>**Review:** Questions to consider for discussion
October 23, 2020	Online<br><br>11:00 – 12:00	**Video 1: Carmen G. Gonzalez: Women of Color in Academia**<br><br>In this video interview Dr. Gonzalez discusses her book *Presumed Incompetent: The Intersections of Race and Class for Women in Academia* (co-edited with Gutiérrez y Muhs and Angela P. Harris, published in 2012 by Utah State University Press). This book is *a 600-page examination of the overlapping roles of class, gender, and race in the professional lives of women faculty of color, with testimony from more than 40 contributors.*<br><br>**Reading:** Turning chutes into ladders for women faculty: A review and roadmap for equity in academia.<br><br>Michelle I. Cardel, Emily Dhurandhar, Ceren Yarar-Fisher, Monica Foster, Bertha Hidalgo, Leslie A. McClure, Sherry Pagoto, Nathanial Brown, Dori Pekmezi, Noha Sharafeldin, Amanda L. Willig, and Christine Angelini.Journal of Women's Health. May 2020.721-733. http://doi.org/10.1089/jwh.2019.8027<br><br>**Review:** Questions to consider for discussion
October 30, 2020	Online<br><br>11:00 – 12:00	**Reading 1: Structural racism and health inequities in the USA: evidence and interventions**<br><br>Bailey D Krieger N Agénor N Graves J Linos N Bassett MT, Structural racism and health inequities in the USA: evidence and interventions, Lancet 389(10077) 8–14 April 2017, Pages 1453-1463. https://doi.org/10.1016/S0140-6736(17)30569-X<br><br>**Reading 2:** Pollution is Killing Black Americans. This Community Fought back.<br>https://www.nytimes.com/2020/07/28/magazine/pollution-philadelphia-black-americans.html<br><br>**Video 1: "**Pollution is Segregated" says the Father of Environmental Justice – Dr. Robert Bullard. https://www.youtube.com/watch?v=gU-D3YkOe-w<br><br>**Review:** Questions to consider for discussion
November 6, 2020	Online<br><br>11:00 – 12:00	**Reading 1: An exploratory study on microaggressions in medical school: What are they and why should we care?**<br><br>Espaillat, A., Panna, D.K., Goede, D.L., Gurka, M.J., et al. An exploratory study on microaggressions in medical school: What are they and why should we care? Perspect Med Educ 8, 143–151 (2019). https://doi.org/10.1007/s40037-019-0516-3

		**Video 1:** Microaggressions in Academia. The New School featuring Dr. Derald Wing Sue. https://www.youtube.com/watch?v=x-ffdh1FsT4<br><br>**Review:** Questions to consider for discussion
November 13, 2020	Online<br><br>11:00 – 12:00	**Reading 1: Queer in STEM: Workplace experiences reported in a national survey of LGBTQA individuals in science, technology, engineering, and mathematics careers**<br><br>Jeremy B. Yoder & Allison Mattheis (2016) Queer in STEM: Workplace Experiences Reported in a National Survey of LGBTQA Individuals in Science, Technology, Engineering, and Mathematics Careers, Journal of Homosexuality, 63:1, 1-27, DOI: 10.1080/00918369.2015.1078632<br><br>**Reading 2:** James, S. E., Herman, J. L., Rankin, S., Keisling, M., Mottet, L., & Anafi, M. (2016). The Report of the 2015 U.S. Transgender Survey. Washington, DC: National Center for Transgender Equality. https://transequality.org/sites/default/files/docs/usts/USTS-Full-Report-Dec17.pdf Executive Summary p.3-17<br><br>**Videos 1-4.** Related videos posted in e-learning group website<br><br>**Review:** Questions to consider for discussion
November 20, 2020	Online<br><br>11:00 – 12:00	• Debrief – What have we learned?<br>• Discussion – What next?
November 27, 2020	Online<br><br>11:00 – 12:00	**No Meeting – Thanksgiving Break.**
December 4, 2020	Online<br><br>11:00 – 12:00	Presentation/Discussion of interim plans moving forward (draft syllabus, etc.)
December 11, 2020	Online<br><br>11:00 – 12:00	Present any finalized plans (syllabus, etc.); discuss next steps

Canvas: Course information, including readings, links to videos and syllabus will be available on canvas at http://elearning.ufl.edu/. You will use your Gatorlink account to log on.
